# Dose response relationship between program attendance and children’s outcomes in a community based weight management program for children and their families

**DOI:** 10.1186/s12889-019-7094-5

**Published:** 2019-06-10

**Authors:** Santosh Khanal, Leah Choi, Christine Innes-Hughes, Chris Rissel

**Affiliations:** NSW Office of Preventive Health, Liverpool Hospital, Locked Bag 7103, Liverpool BC, NSW 1871 Australia

## Abstract

**Introduction:**

The Go4Fun program in New South Wales, Australia is a community based weight management program for overweight and obese children aged 7–13 years and their families. This study assessed the impact of the number and type of sessions attended on body mass index (BMI) z-score, fruit and vegetable intake and physical activity and sedentary behaviours to determine the number of sessions required to achieve optimal program outcomes.

**Methods:**

Secondary analysis was conducted on pre and post participant program data collected over 3.5 years. Relationships between session attendance and program outcomes were assessed using Spearman’s correlation and multivariate analyses of variance and multivariate regression. Number of sessions required to achieve optimal program outcomes was determined using piecewise linear regression.

**Results:**

For 3090 participants (48.5% of registrants) who attended at least five sessions, outcome measures improved significantly at post program compared with pre (*p* < 0.01). No relationships were seen between number and type of sessions attended and outcome measures.

Children of mothers without a post-school qualification (university degree or vocational qualification) were more likely to achieve lower levels of improvements in BMI z-score (*p* = 0.02) and vegetable intake (*P* < 0.01) than those children with post-school qualified mothers (F = 3.68, *p* = 0.03). Children of mothers without post-school education that attended seven sessions or more achieved significantly better BMI z-score outcomes (*p* < 0.01) than those who attended fewer sessions.

**Conclusions:**

Maternal educational attainment influences program attendance and health and behavioural outcomes in a family based obesity treatment program.

**Electronic supplementary material:**

The online version of this article (10.1186/s12889-019-7094-5) contains supplementary material, which is available to authorized users.

## Background

Childhood obesity can lead to lifelong overweight and obesity with adverse health effects throughout life. A recent analysis [1] of worldwide data has shown that childhood obesity rates have plateaued in developed countries, albeit at high levels, and are increasing in developing countries. A concerted approach is required to tackle childhood obesity that includes community based programs to manage childhood obesity, interventions in schools and other settings and media campaigns to promote healthier lifestyles and policy changes to influence health behaviours [2]. Community based interventions at the family level are one of the most effective in managing childhood obesity [3] and a systematic review found them to be more effective than school based interventions for children under the age of 12 years [4].

The Go4Fun program in New South Wales (NSW), Australia, described elsewhere [5, 6], is a family based community weight management program for overweight and obese children aged 7–13 years that is delivered once per week [7]. It is a mandatory requirement of the program that a parent or a carer attend the program sessions with their children. The program is a community-based translation of the MEND (Mind Exercise Nutrition Do it) program in the UK which is delivered in association with clinical services [8]. It has since been further modified to adapt to Australian settings and in response to ongoing quality assurance and research findings [5, 7]. The most significant modification was changing program delivery from twice per week to once per week to reduce participants burden and service costs. A cluster randomised controlled trial showed that the condensed once per week version was as effective as twice per week version of the program in achieving health and behavioural outcomes [7]. No program changes were made to the once per week version of the program for the purpose of the current study.

Understanding the level of attendance that is likely to yield optimal participant outcomes would help program staff develop strategies to encourage participants to attend at least the minimum number of sessions that is likely to deliver beneficial lifestyle and health outcomes. The importance of assessing the dose-response relationship between attendance and outcomes for programs targeting childhood obesity has been highlighted previously [9], but it has also been acknowledged that dose-response relationships are difficult to discern in controlled studies because of limited sample size and variability in data [10].

In this paper, using routinely collected pre and post program data for the Go4Fun program between July 2013 and December 2016, we describe the impact of the number and type of sessions attended on BMI zscore and nutritional behaviours at program completion to determine the number and type of sessions likely to generate optimal program outcomes.

## Methods and subjects

Secondary analysis was conducted on routinely collected de-identified Go4Fun program data, which includes anthropometric measurements of the participating child and the attending parent and parent reported nutritional, physical activity and sedentary behaviours. Go4Fun program is delivered over 10 weeks during school terms (January to December, with four terms). Groups typically comprise of 8–12 children. The program is delivered by the local health districts (LHDs), geographical administrative regions for health services in NSW.

All program participants for whom pre and post data were available between July 2013 and December 2016 were used for this study, which includes data for 265 children from a pragmatic incentives study [11] to assess the effectiveness of incentives to families in improving program participation and outcomes (results not yet published). No changes were made to the program structure and delivery approaches for the incentives study.

### Program and participants

Families were enrolled into Go4Fun via the usual enrolment pathways such as self-referral (the majority) or by referral from their health practitioners, and each eligible child was allocated to a local program based on availability and the family’s preferences for day and time. BMI was calculated from the height and weight recorded by the program facilitators who were trained to conduct the measurements using a standardised approach [12]. BMI zscore was calculated using the LMS method described by the Centers for Disease Control [13]. Children were eligible to participate in the Go4Fun program if their pre-program BMI was equal to or more than the 85th percentile for their age on the Centres for Disease Control BMI chart for children [14].

The Go4Fun program at each site was delivered by LHD allocated facilitators who had undertaken the standardised Go4Fun training as a prerequisite for employment as a facilitator [15]. As part of the program leader training, the program leaders are trained on program related anthropometric measurements and questionnaire administration. A site requires a minimum of five registered participants for the program to run. Children were typically enrolled a few weeks before program commencement and it was a mandatory requirement that a parent or carer attended each session with their child.

### Measurements

Pre and post program measurements were conducted by the trained program facilitators.

The attending parents completed a questionnaire on physical activity, sedentary activities and dietary behaviour that had been used previously [7]. Parent questionnaire items were from a previously validated parent reported dietary questionnaire for children aged two to five years [16] and self-reported physical activity [17] and sedentary behaviour [18] questionnaires for adolescents. The self-response phrasing of the physical activity and sedentary behaviour questions were modified for parent response without changing the questions and the response items. Physical activity items relate only to physical activity outside the Go4Fun program.

The dietary questions included the number of servings of vegetables, fruit and other food types (mostly energy dense nutrient poor food) as well as the number of cups of water and other drinks such as milk and soft drinks the child consumed. For the purpose of this study, the vegetable and fruit intakes were assessed because fruit and vegetable intake has been demonstrated to be a reliable measure of childhood obesity [19] and there are specific guidelines in Australia for fruit and vegetable intake in children [20].

Questions asked about the total amount of time (hours and minutes) during the week the child walked and participated in moderate to vigorous physical activity during the week for recreation, exercise or to get to or from places, with the response option an open field for the number of times. Questions about sedentary time asked about the usual weekday, and each of the weekend days, how much time the child spent sitting and using a mobile phone, iPad, tablet, computer, gaming console or watching TV/DVDs. Total weekly duration of physical activity was determined by adding the time spent participating in organised sports and non-organised physical activity. Weekly sedentary behaviour questions were on recreational screen time and sedentary travel modes. The pre-program parent questionnaire also included demographic questions including the highest qualification of mother to represent socioeconomic status, adapted from the Australian Census [21].

For health outcomes, physical measurements were height using a height measuring rod (HM200P, Charder Medical, Taichung City, Taiwan) and weight using a digital scale (Seca clara 803, Seca, Hamburg, Germany). Height and weight were each measured twice and the average taken. Weight was measured in KG and height in CM. Waist circumference was also measured, but these data was not analysed due to concerns raised recently in Australia [19] and UK [9] regarding its reliability in children particularly when it could not be ascertained that pre and post program measurements were conducted by the same program facilitator [22].

All data were entered into a central database, which was also used by program staff to record attendance at each session. BMI z-score at post program and follow up were treated as missing values if the difference score for BMI at post program was ±5 kg/m^2^ from pre-program similar to the approach used in the routine Go4Fun program reporting as it is highly improbable for a child to achieve a BMI change of ±5 kg/m^2^ in a 10 week period. Program sessions were categorised into knowledge and application types by LC and face validated by CIH. (Table 1).

**Table 1:** Outline of the weekly sessions for the once per week Go4Fun programs. Sessions in bold italics are attended by the parents/carers and the children together. In the physical activity component, children participate in games and skills based activities to develop confidence and skills to improve participation in regular physical activity. Sessions were categorised into knowledge or application based on the session contents

Session week	Session type	First hour		Second hour	
		Parents/carers	Children	Parents/carers	Children
1	Measurement	***Meet the leaders and growth check 1***		***Introduction to the program***	
2	knowledge	***Introduction to health and nutrition***		Goals and rewards	Physical Activity
3	knowledge	***Setting family goals and rewards***		Be a moving and grooving family	Physical Activity
4	knowledge	***Refined*** **versus** ***unrefined carbohydrates***		External triggers	Physical Activity
5	knowledge	***Fats and sugars***		Internal triggers	Physical Activity
6	application	***How to read a food label***		***Food label practical (supermarket tour)***	
7	knowledge	***Ready steady, eat***		Bullying	Physical Activity
8	application	***Survival guide to parties, eating out and other tempting occasions***		Parental role modelling and sleep routines	Physical Activity
9	application	***Health and nutrition quiz game, program revision***		Problem solving	Physical Activity
10	Measurement	***Healthy Growth check 2***		***Graduation ceremony***	
Post program		***Group reward***		***Group reward***	

Data collected between July 2013 and Dec 2016 were used to assess the dose response between attendance and the key program outcomes (BMI zscore, fruit and vegetable intake, physical activity, sedentary behaviour) and to assess whether the session type had any effect on program outcomes.

### Statistical analysis

The data were initially analysed descriptively to explore the demographic and other characteristics of participants and their families. Pre and post program date were compared (using a *p*-value of *p* < 0.01 for statistical significance for all analyses), and correlations between the number of sessions attended and the program outcomes (BMI z-score, vegetable and fruit intake, physical activity, sedentary behaviour) were assessed using a boxplot and Spearman’s correlation coefficient to account for the potential monotonic relationship between the variables. An initial multivariate analysis of variance and a subsequent multivariate regression was conducted for the BMI zscore program outcomes as the dependent variable, and the number of sessions attended and the participant and family characteristics as independent variables.

As a post-hoc analysis, for the participant characteristic that was found to be statistically significant on the multivariate analyses, a line graph with interpolation was plotted for BMI z-score for all mothers against the number of sessions attended to determine a cut-off for the number of sessions until which incremental outcomes were achieved. Independent t-test was conducted to compare the BMI zscore outcomes for participants with and without university qualifications attending fewer or more sessions than the cut-off. A piecewise linear regression using the cut-off as the break point was then conducted as a sensitivity analysis to confirm the cut-off.

We would expect the ICC for obesity and overweight to be high because we have recruited only from the top 15 percentile of the BMI distribution. We have not used multi-level modeling in this paper because in our previous (published) RCT (comparing once a week with twice a week delivery) we did not find any differences when the analysis was done at the group and Local Health District levels [7]. An initial analysis with Local Health District in the current data did not find any effect and was dropped from consideration.

## Results

A total of 6366 eligible children registered for the program within the study period of which pre-program data were available for 5389 participants (84.7% of registrants) and post-program data for 3090 participants (57.3% of those with pre-program data). Program delivery occurred at 426 sites, with the number of participants ranging from four to 22. The intracluster coefficient (ICC) for BMI z-scores was found to be 0.13.

The average age of the children who registered for the program was 9.8 (±1.9) years, 50.6% of whom were females (see Additional file 1: Table S1). Most participants were brought to the program by their mothers (*n* = 4327, 72.6%). Children who did not attend the post-program measurement session attended 4.2 (±2.3) sessions on average whereas the children for whom the post program data were available attended 8.8 (±1.4) sessions on average. The children who completed the Go4Fun program were more likely to be from families with post-school qualified mothers and those that lived with more than one parent or carer. There were no differences in the other demographic characteristics and the health and behaviourial measures between the children who attended or did not attend the post program measurements (Additional file 1: Table S1).

Children with post program data who attended fewer than five sessions (*n* = 38) were excluded from further analysis as the low numbers did not allow a reliable assessment of the association between attendance and outcomes. For participants who attended at least five sessions, there was a statistically significant change in BMI z-score and other outcome measures at post program compared with pre (*p* < 0.01 for all outcome measures) (Table 2). The number of sessions attended showed weak correlations with BMI z-score and fruit and vegetable intake and no correlations with time spent on physical and sedentary activity (Table 2).

**Table 2:** Pre and post program outcome measures. *P* values were generated from the multivariate analysis of variance for the outcome measures and the correlation coefficient (r) is the Pearson’ correlation between the difference in pre and post outcome measures and the number of sessions attended

*N* = 3662	Pre program	Post program	*P* value	Correlation coefficient (r)
BMI zscore	1.87 (0.52)	1.77 (0.56)	< 0.01	−0.06
Veg intake (serves /day, SD)	1.4 (0.9)	2.0 (1.1)	< 0.01	0.12
Fruit intake (serves /day, SD)	1.8 (0.9)	2.0 (0.8)	< 0.01	0.06
Physical activity (hrs/wk., SD)	3.3 (2.0)	4.0 (2.0)	< 0.01	ns
Sedentary activity (hrs/wk., SD)	22.7 (13.3)	18.3 (10.7)	< 0.01	ns

The multivariate analysis of variance (F = 3.68, *p* = 0.03) and the subsequent multivariate regression showed that the only variable likely to influence the BMI zscore was highest qualification of mother. (Table 3). Children of mothers without university degree qualifications were likely to achieve significantly lower levels improvements in BMI zscore (β =0.02, *p* < 0.01) at program completion compared to the children of mothers who were university degree qualified. No effects were seen for number and type of attended sessions, age and gender of child, parent BMI at pre-program and household type.

**Table 3:** Regression coefficients and the significance levels for predictors of BMI zscore change at post program measurement compared to pre from the multivariate regression

Predictor	Coefficients (β)		*p* value
	Unstandardised (Std. error)	Standardised	
Gender	.010 (0,01)	0.03	0.17
Language other than English spoken at home	−0.10 (0,01)	−0.02	0.25
Mother with university qualification^a^	.02 (0,01)	0.06	< 0.01
Socioeconomic status	0.01 (0,01)	0.04	.0.08
Total knowledge sessions attended (Session numbers 2,3,4,5,7)	--0,01 (0,01)	−0.01	0.82
Total application sessions attended (Session numbers 6,8,9)	−0.01 (0,01)	− 0.03	0.45

^a^ represents a statistically significant predictor of BMI zscore decrease at post program

### Post-hoc analysis of children of mothers with and without university degree qualification

**Fig. 1:**
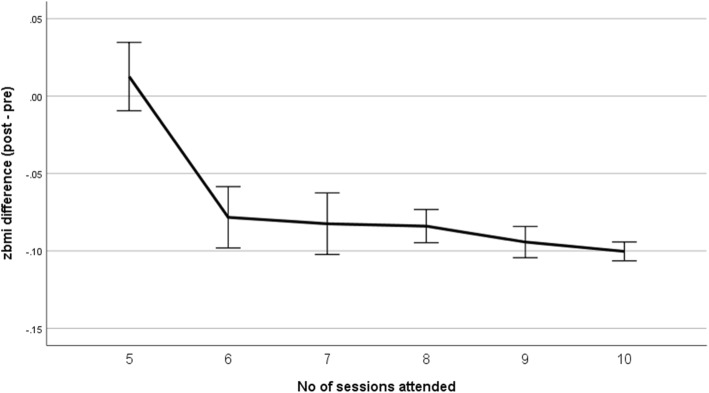
Change in zbmi score at program completion from program start (post – pre) by the number of sessions attended for all participants (*N* = 3052). The error bars represent 95% confidence intervals

**Fig. 2:**
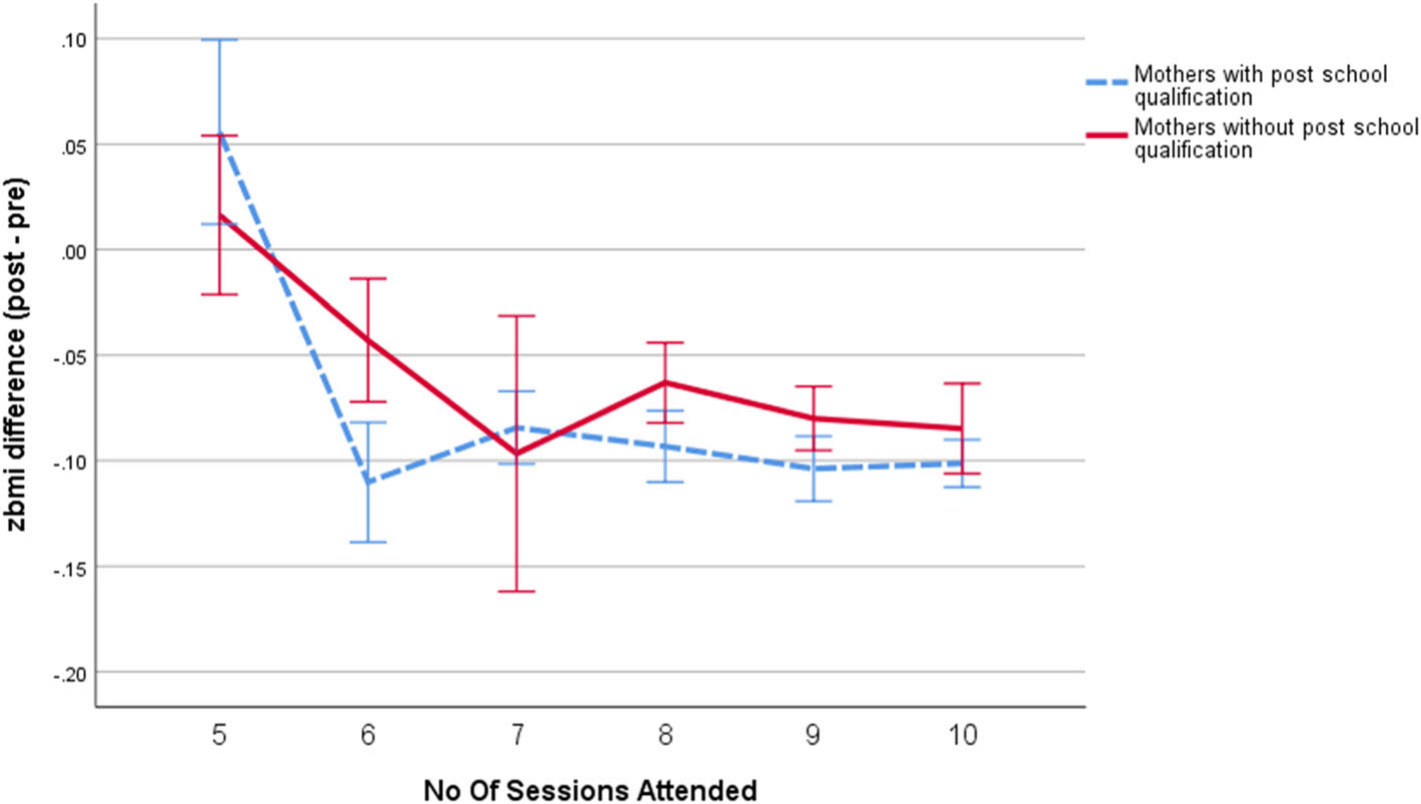
Change in zbmi score at program completion from program start (post – pre) by the number of sessions attended for children of mothers with (*N* = 1925) and without post-school qualifications (*N* = 1127)

The mean difference in BMI z-score outcomes was statistically significant when compared by seven sessions or more (− 0.8 ± 0.1) or less than seven sessions (− 0.5 ± 0.1) (*p* < 0.001). However, there does not appear to be incremental benefits in BMI z-score outcomes of attending more than seven sessions (Fig. 1). This finding was confirmed by the intercept (≥7 sessions) of the piecewise regression (β = − 0.76, t = − 0.68, *p* < 0.01). In comparison, children of mothers with post school qualifications were able to achieve a BMI z-score change of − 1.0 after attending six sessions (Fig. 2).

At least seven sessions were attended by 55.7% of all children with mothers without a university degree qualification, which was slightly lower than the proportion of children of university educated mothers who attended at least seven sessions (63.9%).

## Discussion

Our study, using routinely collected data from large scale routine implementation of the Go4Fun program, supports previous reports that participants of the scaled up childhood obesity programs based on the MEND program achieve positive health and behaviourial outcomes at program completion [22–25]. Our study adds to the evidence that large-scale weight management programs for families of overweight and obese children are a useful tool in tackling obesity in children, in line with findings from previous reports of MEND based and other interventions [3, 22–26].

An important finding in our study was the association between maternal educational level and program outcomes. Participants that had mothers without university qualifications benefited incrementally from attending more sessions until seven sessions after which the impact of the program plateaued, whilst other participants seem to achieve better BMI z-score outcomes by attending fewer sessions. It has been reported in other areas of health care that families with low level of parental literacy are less likely to fully participate in or adhere to programs like Go4Fun [27, 28] and achieve worse health care [29] and program outcomes [30] which subsequently lead to health inequities [31]. Specifically for the Go4Fun program, strategies need to be developed to encourage families with low levels of parental literacy to attend at least seven sessions. Families who speak a language other than English at home may also benefit, but our analyses did not find language spoken at home was associated with attendance. This target should be achievable as more than half of the families with mothers without university degree qualifications in Go4Fun already attend at least seven sessions.

There was no distinctive pattern in our study of participants attending either a session which was based on learning information or obtaining knowledge e.g. a session learning about fats and sugars or a session around application of knowledge e.g. supermarket tour. Session type did not influence the program attendance or outcomes for Go4Fun families. This may be because attendance is often determined by factors outside of the program schedule (for example, illness, or competing time demands) and material covered in the sessions can be caught up or learned from the program resource material. Interest in program content is one of the main predictors of attrition in paediatric weight management programs [32, 33] along with other competing priorities and logistical factors [33]. From a program implementation perspective, this means that it is crucial to ensure participants remain interested in the overall program content area but it may not be necessary to prioritise some sessions over others.

In a broader context, our finding highlights the strengths of routinely collected robust program data in monitoring and evaluating weight management programs delivered at scale to identify program implementation strategies that can be key influencers of program outcomes [34]. Conversely this is not usually possible with experimental data due to insufficient sample size. Nonetheless, the use of routine program data for assessing outcomes and using clustered data have limitations. Although recent studies have shown that measurements by trained staff are sufficient for research purposes and are reliable [35, 36], we could not validate the accuracy of the collected anthropometric data. Further, self-reported/parent-reported physical activity, sedentary behaviour and dietary intake has some limitations, as these behaviours can be difficult to recall. However, child recall of these behaviours is very poor, and at least the parents are able to report consistently (at the beginning and end of the program) about what they were able to observe. The ICC in our study was considerably higher than the values reported for other prospective clinical and population based studies with cluster designs. The high ICC for BMI zscore in our sample is perhaps not surprising given the mandatory program requirement that all participants need to be overweight or obese. Nevertheless, the high ICC reinforces the need for large sample sizes when assessing program outcomes.

Given the large sample size however, it can be expected that the effect of any measurement errors in the final outcomes is likely to be small. Also, it is not possible from our study to establish cause-effect associations for program outcomes, and we could not ascertain the reasons for families withdrawing from the program. Despite these limitations, we have been able to gain insights into how the program benefits can be optimised for participating children.

Our study has been able to show that children from families with mothers without university degree qualifications need to attend more program sessions than those with university degree qualified mothers to achieve similar level of health and behaviourial outcomes in family based weight management programs.

## Additional file

Additional file 1: **Table S1.** Comparison of demographic characteristics and health and behaviourial measures at pre program of children for whom post program data was not and was available. (DOCX 14 kb)

## Data Availability

Data are available from the authors.

## Abbreviations

BMI: Body mass index
CM: Centimetres
KG: Kilograms
NSW: New South Wales
