# Supplementary material for: Dose response relationship between program attendance and children’s outcomes in a community based weight management program for children and their families
Source: BMC Public Health. 2019 Jun 10;19:716. doi: 10.1186/s12889-019-7094-5 (PMC6558714; doi:10.1186/s12889-019-7094-5)
Supplement: Supplementary file 1 — Table S1. Comparison of demographic characteristics and health and behaviourial measures at pre program of children for whom post program data was not and was available. (DOCX 14 kb) [file 12889_2019_7094_MOESM1_ESM.docx]

Additional file 1: Table S1 Comparison of demographic characteristics and health and behaviourial measures at pre program of children for whom post program data was not and was available

| Pre program measurements | Participants without post program data  (N=2299) | Participants with post program data  (N=3090) |
| --- | --- | --- |
| *Demographic characteristics* | | |
| Average age (yr±SD) | 9.8 (1.8) | 9.8 (1.8) |
| Females (%) | 48.5 | 50.6 |
| English not spoken at home (%) | 45.8 | 49.7 |
| Mothers with health care card (%) | 39.9 | 38.3 |
| Mothers without post-school qualifications (%)* | 28.9 | 22.1 |
| Sole parent household (%)* | 25.4 | 17.7 |
| *Health and behaviourial measures* | | |
| BMI zscore (±SD) | 1.9 (0.6) | 1.9 (0.5) |
| Fruit intake (serves/day ±SD) | 1.7 (0.9) | 1.8 (0.9) |
| Vegetable intake (serves/day ±SD) | 1.4 (0.9) | 1.5 (1.0) |
| Sedentary activity (hours/week ±SD) | 23.1 (14.9) | 22.6 (13.4) |
| Physical activity (hours/week ±SD) | 2.7 (2.0) | 3.3 (2.0) |

*Differences statistically significant (p<0.01)
